# Upadacitinib for recalcitrant extraintestinal Crohn disease

**DOI:** 10.1016/j.jdcr.2025.11.031

**Published:** 2026-01-17

**Authors:** Avery H. Seward, Jeffrey P. Callen

**Affiliations:** a Eastern Virginia Medical School at Macon & Joan Brock Virginia Health Sciences at Old Dominion University, Norfolk, Virginia; b Department of Dermatology, University of Louisville School of Medicine, Louisville, Kentucky

**Keywords:** extra-intestinal Crohn disease, upadacitinib

## Introduction

Crohn disease is a chronic inflammatory bowel disease that affects the gastrointestinal tract from the mouth to the anus. Extra-intestinal manifestations of Crohn disease are common, with the skin being the most commonly involved site.^1^ Cutaneous manifestations of Crohn include lesions that directly extend from the bowel to the skin, whereas extraintestinal Crohn disease involves sites that do not directly extend from the bowel.^1^ Extraintestinal Crohn disease is often resistant to treatments and often does not parallel the activity of the bowel disease.^1^ There have only been 3 documented cases of extraintestinal Crohn disease responding to upadacitinib.^2^^,^^3^ We describe a patient with recalcitrant extraintestinal Crohn disease who responded well to upadacitinib allowing her to cease prior therapies.

## Case

**Fig 1:**
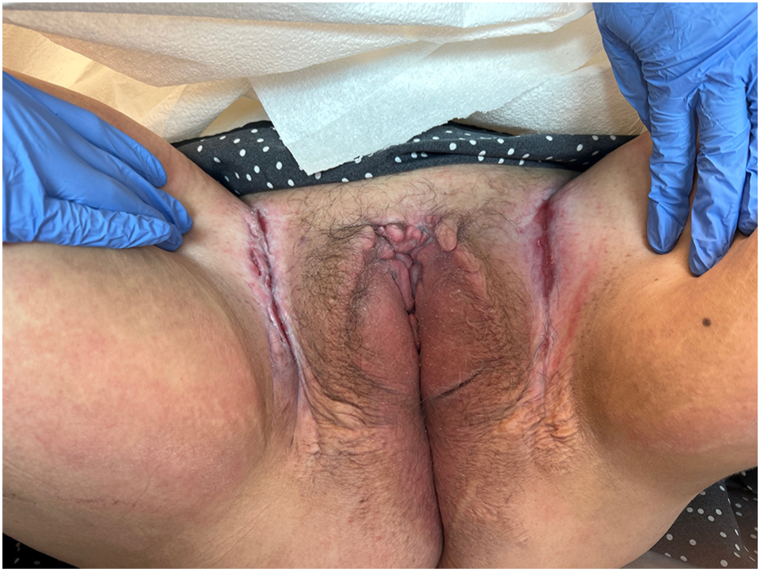
Knife like ulcerative lesions of patient’s inguinal folds and hypertrophy of the vulva and clitoris prior to the introduction of upadacitinib.

A woman in her late 30s with a 29-year history of extraintestinal Crohn disease, with past medical history of an ileostomy pouch following a total colectomy, developed severe cutaneous Crohn disease affecting her perineum that included knife-like ulcerative lesions of her inguinal folds and hypertrophy of the vulva and clitoris (Fig 1). For the past 12 years as our patient, these manifestations have been resistant to multiple pharmacotherapies, including metronidazole, ustekinumab, mycophenolate mofetil, and methotrexate.

Biopsy of gluteal and inguinal lesions revealed focal granulomatous infiltrate with background lymphoplasmacytic inflammation with small foci of granulomatous inflammation consisting of a few multinucleated histiocytes and a few mononuclear histiocytes. These findings were supportive of a diagnosis of extraintestinal Crohn disease.

**Fig 2:**
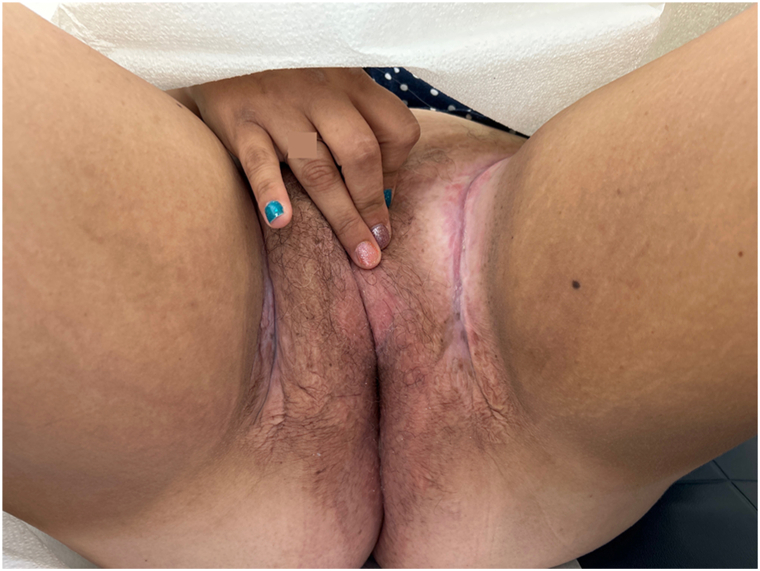
Six weeks of upadacitinib 45 mg daily demonstrated improvement of patient’s ulcerative slit-like lesions of the inguinal folds, with only mild erythema remaining.

Given the patient’s resistance to previous trialed pharmacotherapy, upadacitinib 45 mg, the recommended dosage for gastrointestinal Crohn disease, was initiated while her current regimen of oral metronidazole 500 mg twice daily, hydrocortisone ointment, silver sulfadiazine, and triamcinolone ointment were continued. Within 6 weeks, improvement was noted. The patient’s ulcerative slit-like lesions of the inguinal folds healed completely with only erythema remaining (Fig 2). Clitoral and vulvar hypertrophy have improved as well. No adverse effects or laboratory abnormalities from upadacitinib have been noted during treatment. We plan to taper the patient’s dose to 15-30 mg after 1 year of remission, based on clinical response.

## Discussion

Extraintestinal Crohn disease is a rare manifestation of Crohn, presenting with cutaneous manifestations that are not contiguous with the gastrointestinal tract.^4^ The presentation of extraintestinal Crohn is variable and can include ulcerations, plaques, and nodules that are commonly found on the moist skin folds, genitals, and extremities.^5^, ^6^, ^7^ Our patient fulfills the criteria for diagnosis of extra-intestinal Crohn, as she has knifelike ulcerations of the inguinal skin, genital swelling, and a diagnosis of inflammatory bowel disease.^8^ The mechanism of extraintestinal Crohn’s disease remains unclear but is postulated to involve a Th1-mediated delayed hypersensitivity reaction.^9^ On biopsy, extraintestinal Crohn disease appears similar to intestinal Crohn, with characteristic findings of “noncaseating granulomas and epithelioid histiocytes” although this is not necessary for diagnosis.^4^ Extraintestinal Crohn disease is often refractory to treatment and causes significant morbidity and discomfort.

There is currently no standard for pharmaceutical management of extraintestinal cutaneous Crohn disease.^4^ Corticosteroids, antibiotics, immunosuppressants, tumor necrosis factor inhibitors, and interleukin 12 of 23 blockers are agents that have shown some efficacy,^3^^,^^6^ although many patients remain refractory to these treatments. Upadacitinib is an oral selective JAK1 inhibitor used to treat many chronic inflammatory conditions including rheumatoid arthritis, atopic dermatitis, psoriatic arthritis, Crohn disease, and ulcerative colitis.^10^ Prior to the initiation of upadacitinib, patients should receive a QuantiFERON-TB Gold test and pregnancy is contraindicated.

Upadacitinib has been used to resolve a case of extraintestinal Crohn presenting with a hand lesion,^2^ as well as 2 cases of extraintestinal Crohn involving the genital regions and inguinal folds.^3^ (Table I).

**Table I:** Reported cases of extraintestinal manifestations of Crohn disease treated with upadacitinib

Patient	Age/sex	Site of extraintestinal manifestations	Duration of lesions	Upadacitinib dose	Time to complete healing/resolution
Burningham KM, et al.	63-y-old man	Hand	8 mo	30 mg → 45 mg daily	4 mo
Ebriani J, et al.	Woman in her 40s	Genital region, inguinal folds	5 y	45 mg daily	2 mo
Ebriani J, et al.	Woman in her 60s	Genital region, inguinal folds	2 y	45 mg daily	3 mo
4 (current case)	Woman in her late 30s	Genital region, inguinal folds	>12 y	45 mg daily	6 wks

Our patient demonstrated rapid resolution of skin lesions despite a longstanding disease course of over 12 years, suggesting that patients refractory to pharmacotherapy for extraintestinal Crohn disease may benefit from upadacitinib.

## Conflicts of interest

None disclosed.
